# Supplementary material for: Tuning electromagnetic properties of SrRuO3 epitaxial thin films via atomic control of cation vacancies
Source: Sci Rep. 2017 Sep 14;7:11583. doi: 10.1038/s41598-017-11856-z (PMC5599527; doi:10.1038/s41598-017-11856-z)
Supplement: Supplementary file 1 — Supplementary Information [file 41598_2017_11856_MOESM1_ESM.pdf]

Supplementary Information

Tuning electromagnetic properties of $SrRuO_3$ epitaxial thin films via atomic control of cation vacancies

Sang A Lee[1], Seokjae Oh[1], Jegon Lee[1], Jae-Yeol Hwang[2], Jiwoong Kim[3], Sungkyun Park[3], Jong-Seong Bae[4], Tae Eun Hong[4], Suyoun Lee[5], Sung Wng Kim[2], Won Nam Kang[1], and Woo Seok Choi[1*]

[1]Department of Physics, Sungkyunkwan University, Suwon, 16419, Korea

[2]Department of Energy Sciences, Sungkyunkwan University, Suwon 16419, Korea

[3]Department of Physics, Pusan National University, Busan 46241, Korea

[4]Busan Center, Korea Basic Science Institute, Busan 46742, Korea

[5]Electronic Materials Research Center, Korea Institute of Science and Technology, Seoul 02792, Korea

[*]e-mail: choiws@skku.edu.

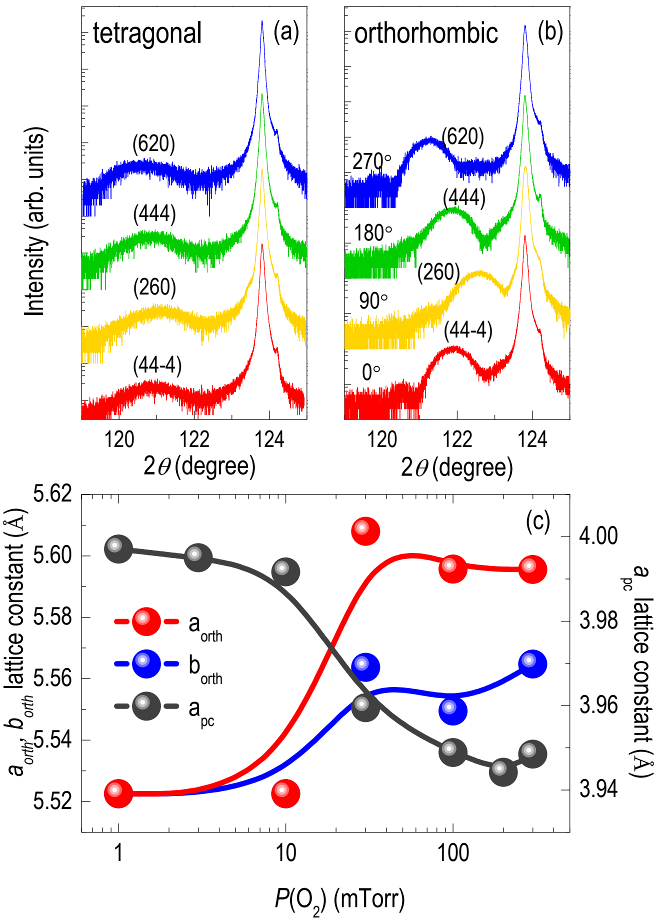

Supplementary Figure S1. Off-axis x-ray diffraction for the orthorhombic and tetragonal $SrRuO_3$ thin films. The $SrRuO_3$ thin films grown at at $P(O_2)$ = (a) 10 and (b) 100 mTorr, around the $SrTiO_3$ (204) Bragg reflections with configuration of φ = 0, 90, 180, and 270°. (c) Evolution of the orthorhombic and pseudocubic lattice constants of epitaxial $SrRuO_3$ thin films as a function of $P(O_2)$.

$P(O_2)$ (mTorr)	a_o (Å)	b_o (Å)	c_o (Å)	γ_o (°)	α_c (°)
300	5.592	5.548	7.810	89.020	89.548
100	5.596	5.549	7.810	88.976	89.524
30	5.608	5.564	7.810	88.709	89.547
10	5.523	5.523	7.983	90	90
1	5.523	5.523	7.986	90	90

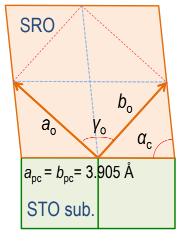

Supplementary Table. S1. Lattice parameters of $SrRuO_3$ thin films grown at various $P(O_2)$. Where a_o, b_o, c_o, γ_o, and α_c are the distorted orthorhombic unit-cell lengths, orthorhombic and pseudocubic tilt angle, respectively. The right side figure is unit cell of $SrRuO_3$ under compressive strain.

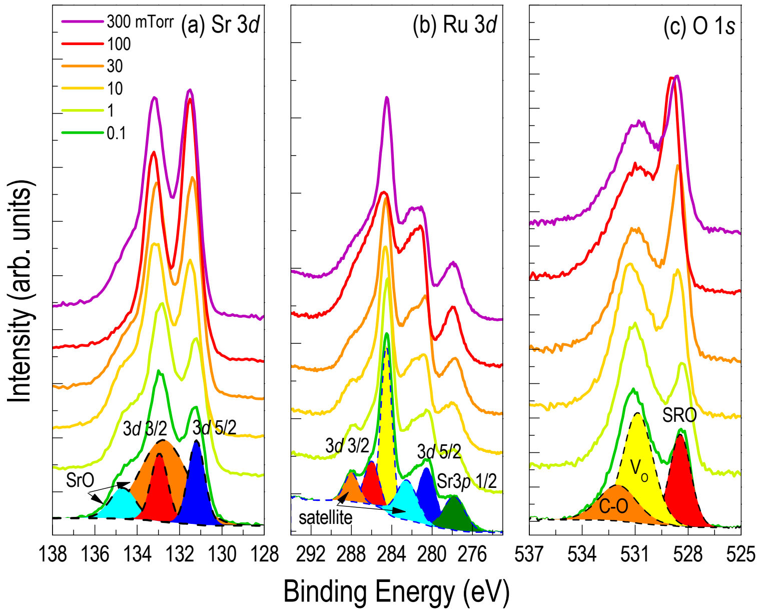

Supplementary Figure S2. X-ray photoemission spectroscopy (XPS) results of (a) Sr 3*d*, (b) Ru 3*d* core-level and (c) O 1*s* spectra for the $SrRuO_3$ thin films grown at different $P(O_2)$.

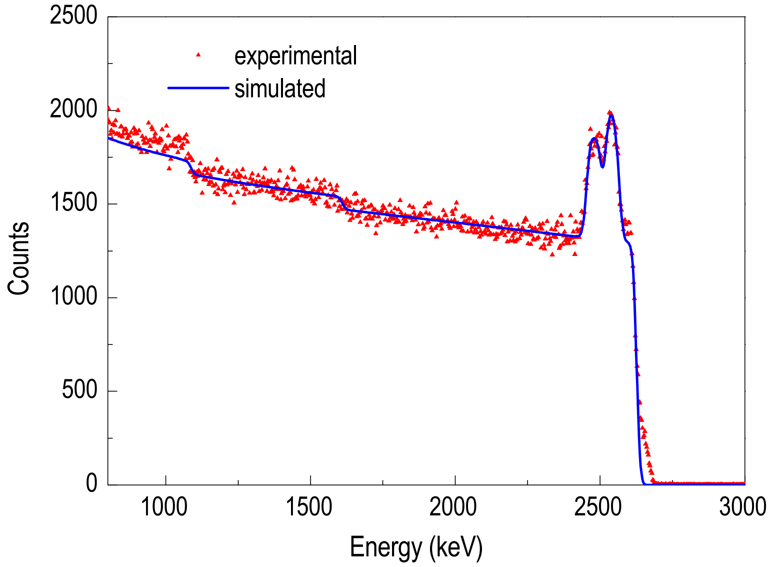

Supplementary Figure S3. The Rutherford backscattering (RBS) data of $SrRuO_3$ thin film.

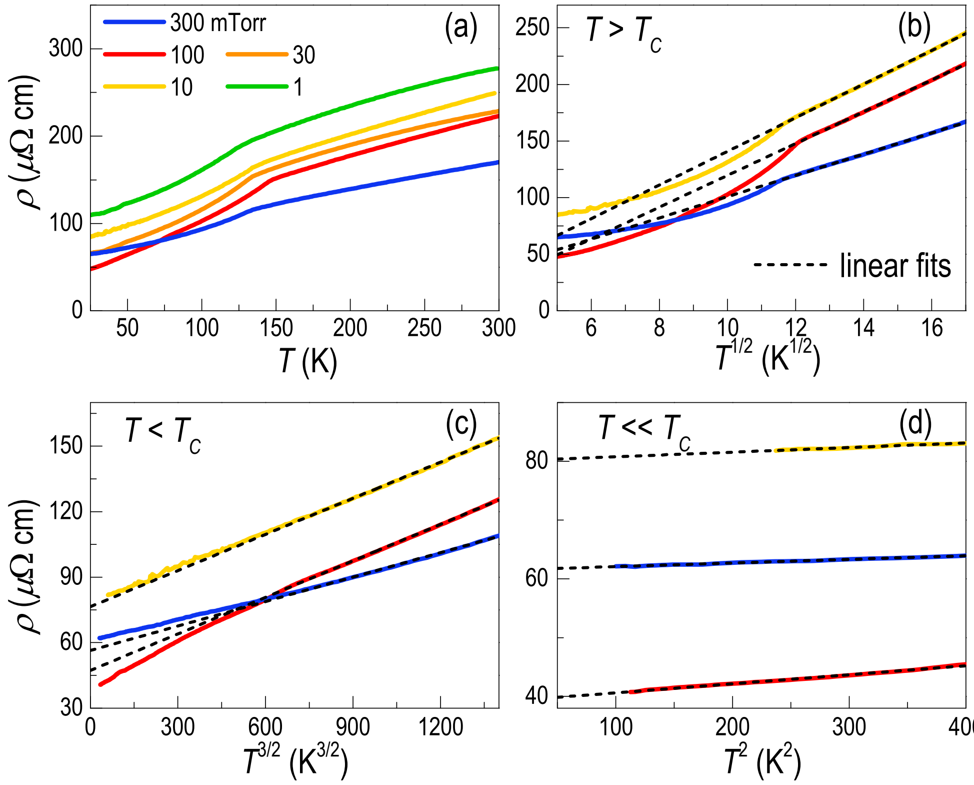

Supplementary Figure S4. Changes in electrical properties of $SrRuO_3$ thin films. (a) Resistivity as a function of temperature ($\rho(T)$) for $SrRuO_3$ thin films deposited at different $P(O_2)$. The resistivity of the $SrRuO_3$ thin films with different $P(O_2)$ are fitted with the power law $\rho(T) = \rho_0 + AT^{\alpha}$ (ρ_0 is residual resistivity, A is temperature-dependent coefficient, α is scaling parameter, and T is temperature) at (b) $T > T_C$, (c) $T < T_C$, and $T << T_C$.

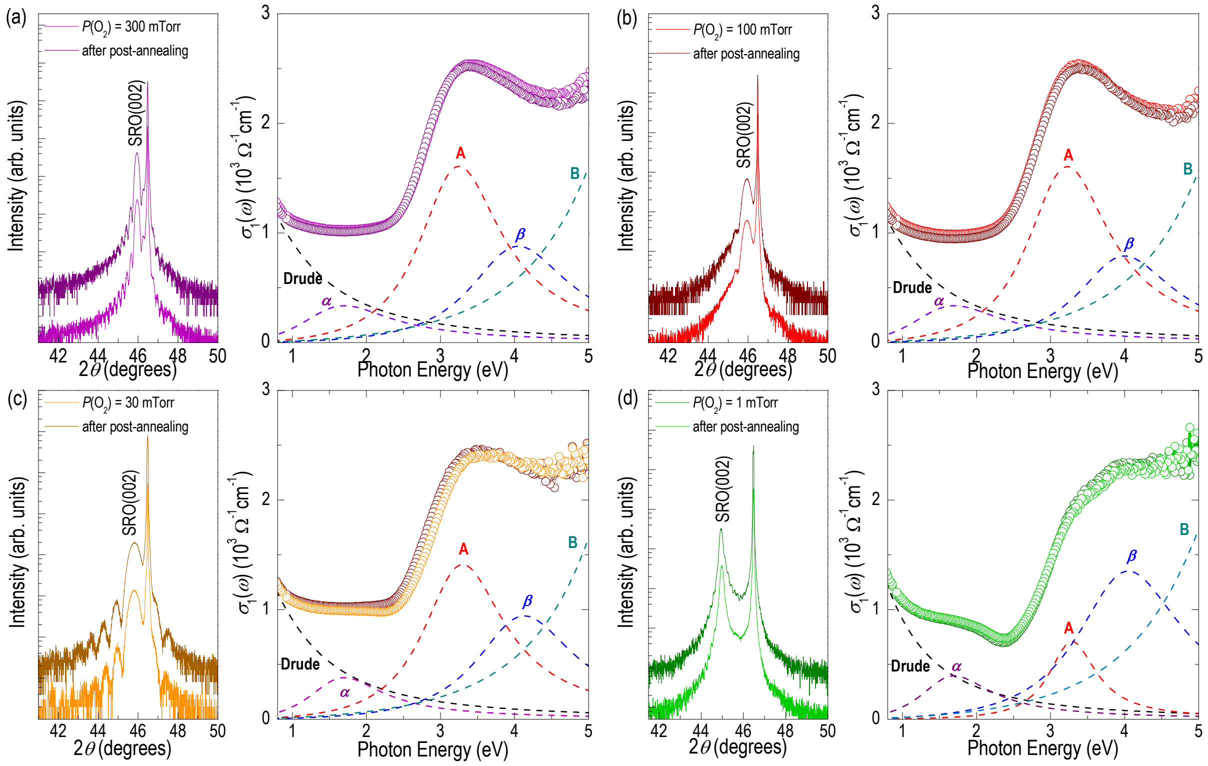

Supplementary Figure S5. Crystal and electronic structure of $SrRuO_3$ thin films. Robust crystal and optical properties of the $SrRuO_3$ thin films with $P(O_2)$ = (a) 300, (b) 100, (c) 30, and (d) 1 mTorr before and after thermal annealing at 700°C for 2 h in air.
